# Clinical proteomic biomarkers: relevant issues on study design & technical considerations in biomarker development

**DOI:** 10.1186/2001-1326-3-7

**Published:** 2014-03-29

**Authors:** Maria Frantzi, Akshay Bhat, Agnieszka Latosinska

**Affiliations:** 1 Mosaiques Diagnostics GmbH, Mellendorfer Strasse 7-9, D-30625 Hannover, Germany; 2 Biotechnology Division, Biomedical Research Foundation Academy of Athens, Soranou Ephessiou 4, 115 27 Athens, Greece; 3 Charité-Universitätsmedizin Berlin, Berlin, Germany

**Keywords:** Clinical proteomics, Biomarkers, Verification, Validation, Mass spectrometry

## Abstract

Biomarker research is continuously expanding in the field of clinical proteomics. A combination of different proteomic–based methodologies can be applied depending on the specific clinical context of use. Moreover, current advancements in proteomic analytical platforms are leading to an expansion of biomarker candidates that can be identified. Specifically, mass spectrometric techniques could provide highly valuable tools for biomarker research. Ideally, these advances could provide with biomarkers that are clinically applicable for disease diagnosis and/ or prognosis. Unfortunately, in general the biomarker candidates fail to be implemented in clinical decision making. To improve on this current situation, a well-defined study design has to be established driven by a clear clinical need, while several checkpoints between the different phases of discovery, verification and validation have to be passed in order to increase the probability of establishing valid biomarkers. In this review, we summarize the technical proteomic platforms that are available along the different stages in the biomarker discovery pipeline, exemplified by clinical applications in the field of bladder cancer biomarker research.

## Introduction

Diseases with high complexity such as cancer are associated with increased incidence rates worldwide. Recent data reveal that approximately 7.6 million deaths caused by cancer occurred in 2008, with this number corresponding to 13% of all deaths [1]. Based on these numbers, there is substantial room for improvement in the current strategies for development of biomarkers capable of being introduced into clinical practice. According to the National Cancer Institute (http://www.cancer.gov/), a biomarker is defined as “*a molecule detected in body fluids or tissues that are associated with a special process (normal or abnormal), a condition or disease*”. Depending on the intended use, biomarkers can be distinguished on the following categories; diagnostic biomarkers which incorporate disease detection, prognostic that represent prediction of the course of a particular disease (e.g. recurrence, progression and survival) and predictive that would allow for prediction of the response to treatment which could be subsequently applied in patient assessment [2-5]. In reference to malignant diseases, diagnosis at late stages generally results in poor clinical outcome [6,7]. The intended use of a cancer biomarker would hence be early stage diagnosis and/or prognosis. Thus, biomarkers that would enable early disease diagnosis are required, together with those that would provide prognostic values in disease status and predict an outcome of an illness prior to any treatment designed. Novel prognostic biomarkers may also help clinicians select an optimal therapeutic strategy for individuals, facilitating determination of the response to a specific treatment type. Successful introduction of biomarkers into routine clinical practice becomes the current motive in this research area and is expected to be beneficial to the patients and in health care systems. Discovering biomarkers is a multi-parameter process [8-10] and applying them to routine practice needs a proper consideration of multiple issues [11]. Additionally, since clinical needs differ among various diseases, biomarker development including discovery, verification and validation cannot be restricted to a single methodology. In this article, we summarize the several challenges related to the biomarker research, as well as the available analytical platforms in the field of proteomics.

## Review

### Pipeline for biomarker development

For more than 40 years translational research in academia and industry has attempted to introduce novel biomarkers with clinical utility to improve the management of diseases, especially those with high social and economic burden such as cancer. Although the analytical platforms have expanded widely, especially in the case of Mass Spectrometry (MS), the vast majority of the published biomarker candidates are not introduced in the clinical practice, due to several issues. The lack of a gold standard can be a drawback since no test with an excellent performance is available to compare with the potential biomarkers. A risk of a biased assessment is thus present. Liu et al. recently addressed this issue by proposing a mathematical formula for covariate adjustment [12]. Several other causative factors have been reported to clarify this discrepancy either related to the disease background or the workflow of biomarker development. Along these lines, several levels of variability can be introduced starting with the disease heterogeneity [13,14], which occurs in complex diseases. Most importantly, the lack of valid biomarkers is often a result of an inadequate set up of the discovery and validation stages [15]. For this reason, a very careful design of the biomarker development is required from the discovery phase to subsequent verification and validation stages. At the same time, concurrent knowledge of the clinical background is needed and a clear target driven from the main clinical needs for the study [16,17].

### Clinical conceptions on the biomarkers study design

**Figure 1:**
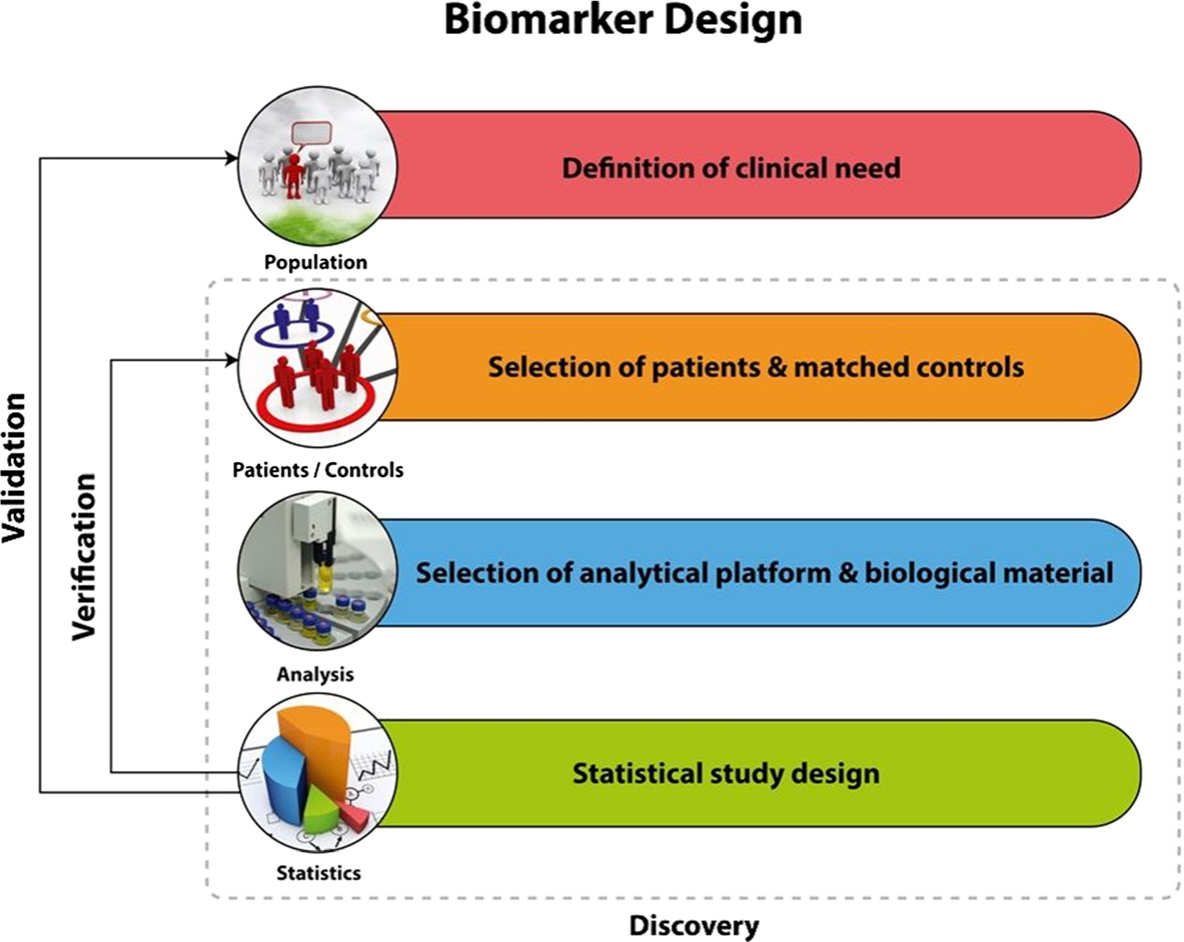
Main components of biomarker study design include definition of clinical need, sample selection and recruitment, statistical evaluation plan and selection of the analytical platform.

A good example to describe relevant issues on variations observed within the same disease is Urothelial Carcinoma. Bladder cancer (BCa) is a highly heterogeneous malignancy characterized by distinct clinical characteristics and molecular pathways [18]. Two independent molecular mechanisms that specifically trigger different phenotypes of urothelial carcinoma have been reported [18]. Ras/MAP kinase signaling activity is specifically involved in superficial phenotypes of the disease. Alterations in tumor suppressor activity of p53 and Rb and overexpression of EGFR and ErbB2, MMP-2 and MMP-9 are characteristic for muscle invasive phenotypes (MIBC) [19-21]. Additional challenges regarding bladder cancer could be that high risk non-muscle invasive BCa (NMIBC) tumour lesions are associated with poor outcome [22,23] and as for the tumor classification the histological variations that can be observed within the same patient as the tumor progresses (intra-patient variation) [24]. These facts should be taken into account in the study design and in terms of clinical objective. The critical parts which have to be considered during the study design are represented in the Figure 1. A biomarker a priori can be applicable only for a specific context of use, for which its performance has been assessed. In the case of bladder cancer, a subgroup or a panel of cancer biomarkers with diagnostic potential that could successfully detect early stage events would be beneficial [25]. A further aim could be the investigation of prognostic indicators for treatment response. The introduction of guidelines in the different biomarker stages has been described as an approach of quality assessment of the biomarkers and standards for designing and reporting biomarker studies have been proposed [26]. For epidemiological studies certain requirements have been suggested in the context of STROBE-ME project [27]. For study design and requirements of predictive biomarkers PROBE standards have been proposed [28], while prognostic biomarkers should be in accordance to REMARK requirements [29].

### Sample biobanking

**Figure 2:**
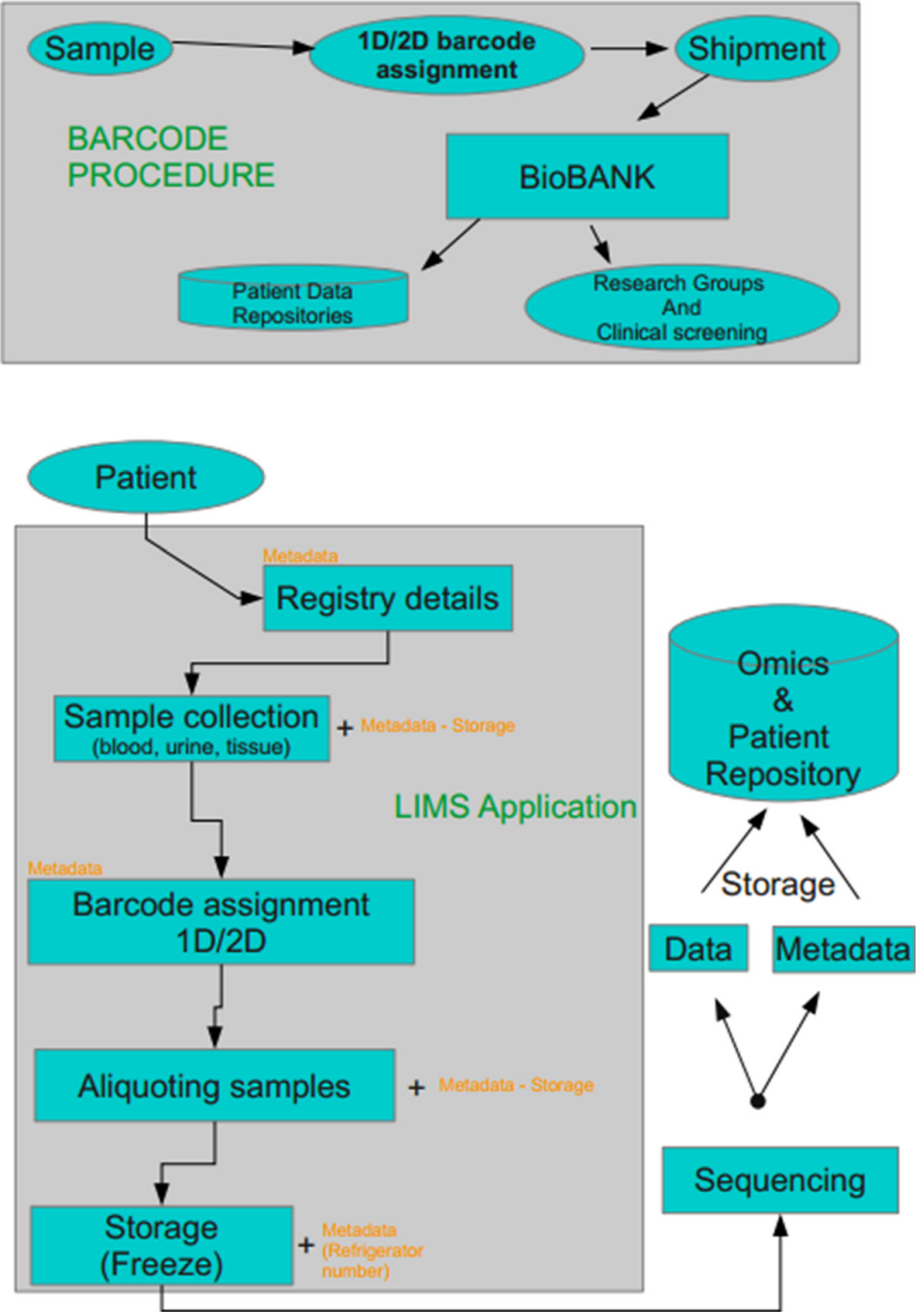
**Representative workflow of the typical procedure to be followed regarding the sample biobanking.** This multistep process includes sample tracking by electronic system, as well as integration of patients clinical characteristics and demographic data. Finally, the deposition of acquired data in public repositories is presented.

To develop a research finding into a clinical tool with diagnostic or prognostic value, a large number of biological samples and/or tissue specimens is required. Prerequisites include not only biological material resources, but also a very well-organized preservation domain to be retained, so called biobank. Ideally, a biobank should retain maximum quality of the biological material stored (following standardized protocols of sample handling), of associated clinical and demographical data, and it should be easily accessible and open to the scientific community [30]. Figure 2 depicts a rough outline of biobanking process [31]. An important issue is assigning a unique ID given to a sample [32], an appropriate database structure and management system. Such systems have been currently developed [33-35] mainly as laboratory informatics management applications (LIMS) that are built to tract samples from the initial steps of delivery.

### Sample collection and treatment

General considerations regarding the variability that can be related to the biological material that is under investigation as well as the sample treatment are also present [36,37]. Two articles published by Lescuyer et al. [38] and Good at al. [39] respectively, address the challenges related to the selection of the biological material for biomarker proteomics applications. In the article by Lescuyer et al. [38], advantages and limitations of the different biological fluids that can be applied in proteomics strategies in search for biomarkers are reported. The biological fluids could be separated into two distinct categories, based on their proximity to the organ of interest. For instance, biological fluids such as CSF, presenting with increased proximity with brain offer the opportunity for defining disease specific biomarkers, although the collection could be in a rather invasive manner. In contrast, in the second category belong body fluids that can be readily available in large amounts, such as urine. The amount of biological material, as well as the non-invasive way of collection, is a major advantage for biomarker studies. The drawback of the analysis of this type of biological fluids could be their heterogeneous content, especially due to the presence of several interfering compounds that are excreted [38]. In the article by Lescuyer et al. the authors also give guidance on selection of samples: especially the inclusion of a reference group that contain both healthy individuals, but also patients with closely related diseases or patients presenting similar symptoms. Further, the performance of the potential biomarkers should not be influenced by pre-analytical factors. To clarify this issue in every type of investigation factors such as storage conditions and proteolysis, for example, should be taken into account and reported [38]. In the follow up article by Good et al. [39], emphasis is put on the selection of the suitable biological fluid or ‘sample source’ as the very first step of the study design. The authors suggest the proper evaluation and reporting of the variability, introduced among others by sampling treatment prior to any analysis [39]. Variability can be limited by the application of approved standard protocols. In bladder cancer biomarker research, urine is a preferred source of biomarkers. Protocols for urine collection and sample processing have been developed by European Kidney and Urine Proteomics (EuroKUP) and Human Kidney and Urine Proteome Project (HKUPP) (http://www.eurokup.org/node/138) [11].

### Study design and evaluation of the analytical performance

Performance parameters of the analytical platform need to be well described and a quality control process must be in place. Recommendations for increased consistency through the application of standardized protocols have been already introduced by McGuire at al. [40] and Fiedler et al. [41]. Guidance may also be gained from a recent manuscript of assessing CE-MS platform performance [42] of the performance of a biomarker is frequently defined by its sensitivity and specificity. Sensitivity in this context is defined as the percentage of the true positive results and specificity to the percentage of true negative results. Skates et al. [43] in their recent study noted the importance of statistical design in biomarker studies. The aim of the above study was the establishment of the method for estimation of the sample size at the initial stage of biomarker development workflow to increase the probability that the selected putative biomarkers will pass the large scale validation in targeted population. The study was focused on the identification of ovarian biomarkers via proteomics approaches including Shotgun analysis of cyst fluids and MRM assay in plasma for discovery and verification, respectively. Therefore, a statistical model was constructed based on the multiple parameters such as distribution of proteins in individual sample, between biological or technical replicates. According to their model an initial cohort of 50 cases and 50 controls could successfully yield a good candidate and if independent verification is applied in a 5 times bigger cohort (250 cases/250 controls), then the chance of a biomarker to pass into clinical validation phase could be >90%. Along the same lines, Shariat et al. [44] reported the need of a well-designed statistical evaluation in the context of defining valid bladder cancer biomarkers, while Behrens and colleagues proposed the validation of bladder cancer biomarkers particularly in prospective studies that meet epidemiological criteria [45]. Collectively, a careful design of the study according to the main clinical needs is required. The initial study is probably best performed in a well phenotyped, predefined cohort and sample handling and analysis should be performed according to strict guidelines. Subsequent mandatory confirmation of the results is best achieved in a prospective multicenter study in the population at risk [46]. Following the above suggestions will substantially increase the possibility that a candidate biomarker be successfully introduced into clinical practice [16,45].

## Technical considerations regarding the analytical set up for biomarker development

**Figure 3:**
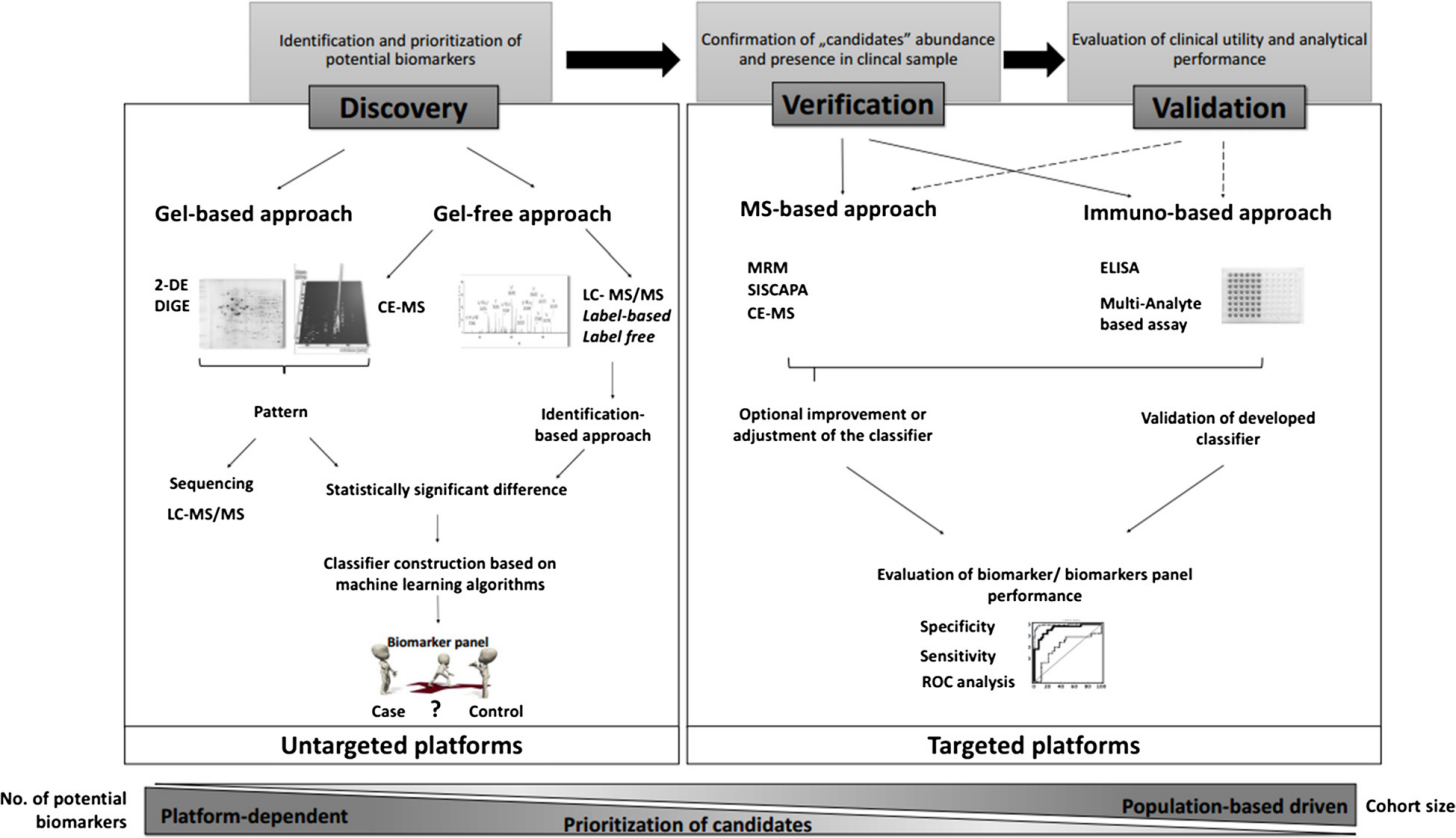
**Schematic representation of proteomics platform applied in biomarker workflow.** Initial discovery phase currently relies on untargeted MS-based approaches resulting in identification of vast number of potential biomarkers. Further verification requires targeted approach. Candidates should to be prioritized based on their functional/ biological relevance. Since the molecular changes underlying the pathological conditions are complex and heterogeneous, the ultimate solution to improve the accuracy of biomarkers appears to be the combination of biomarkers into a panel. The biomarker panel is evaluated in the verification step and further tested during the validation. Currently, immune-based approached are most commonly applied, although moderate selectivity of antibodies represents a significant problem. Alternatively, quantitative MS-based approach like MRM can be also introduced. Along with the advancements in biomarker workflow, the number of putative biomarkers is often decreasing, whereas the sample sets and general costs are increasing. In the validation phase, biomarker performance has to be assessed in a large cohort study in targeted population.

The complexity of the biological fluid may generate a need for a combination of different techniques such as fractionation approaches. In general, the basic requirements of the methodology that is selected are: simplicity of use, robustness, high accuracy and performance [47]. The biomarker workflow can be divided into 3 main parts: discovery, verification and validation. Depending on the specific aim of the study, various proteomic platforms can be applied from the unbiased discovery setting to the targeted quantification in the verification and validation stages. The basic characteristics of the objectives and platforms that can be employed at the different stages are summarized in the Figure 3. Below, a technical description of the available proteomic technologies for discovery, verification and validation stages is presented, together with certain recent applications particularly in the field of clinical proteomics in the quest of bladder cancer biomarker research.

### A) Discovery of biomarkers

Identification of biomarker candidates is the first step towards clinical implementation [48]. At the discovery phase, two major approaches can be distinguished: a knowledge-based approach wherein selection of biomarker candidates is based on the existing molecular mechanisms underlying the disease initiation or progression, or alternatively an unbiased approach that involves untargeted identification of differentially expressed proteins between two analyzed groups [48]. Currently, MS-based proteomics techniques favor untargeted approaches in biomarker discovery that result in a substantial increase of novel biomarker candidates [49,50]. However, due to the limited number of analyzed samples, a high false discovery rate is observed and the reported differential expression frequently are a result of inter- or intra- individual variability. For example, 10% of urinary proteome is unique for a particular individual [51]. Small sample size at discovery phase leads to overestimation of accuracy of biomarker performance (i.e. sensitivity and specificity) and brings the reliability of findings into question [52]. Consequently, the confirmation of detected differences and further investigation of the impact of related diseases on a biomarker’s performance is mandatory.

In general, the classical proteomics workflow includes protein separation using gel-based or gel free techniques followed by the identification by mass spectrometry. Issues that have to be taken into account include establishment of well characterized procedures and platforms along with assessment of analytical variability in addition to the resolution being appropriate for the complexity of the analyzed material [52]. The most common specimen sources for biomarker discovery are body fluids (e.g. plasma, urine and cerebrospinal fluids) that are characterized by high complexity and wide dynamic range in protein concentration. In the case of plasma, the dynamic range is exceeding 10 orders of magnitude [53]. Despite recent advances in the MS-based techniques, the typical MS-based analysis covers only the dynamic range of three to four orders of magnitude [54]. Since the potential biomarker candidates often belong to the low abundance proteins their detection is hampered by the presence of the highly abundant proteins. This is especially evident in plasma where more than 95% of the total protein content comes from only 5 abundant proteins. In order to “dig the deep proteome” and increase coverage, several methods can be applied. One of the recommended strategies is reducing the sample complexity. This approach can be applied at several stages of the experimental workflow. Samples can be enriched for selected subcellular fractions or for specific type of proteins (based on post-translational modifications). Particularly, membrane ([55,56]), secreted [57,58], nuclear matrix [59] and phosphorylated proteins [60] and glycoproteins [61,62] are reported as a valuable source for biomarker identification. Moreover, in the case of plasma, depletion of highly abundant protein like albumin and IgG is widely applied. Several methods have been developed in order to improve the detection of proteins in the low concentration range. Comparison and/or evaluation of different depletion methods was a subject of several studies [63-66]. As an example Tu et al. [66] compared the protein content from plasma samples depleted using immunoaffinity chromatography with unfractionated samples. Applied methods (Multiple Affinity Removal System, Agilent Technologies, Inc.) enables removal of 7 or 14 most abundant plasma proteins. IEF-LC-MS/MS analysis revealed enrichment for 23 low abundant proteins in depleted fraction, which covered around 5-6% of total protein identifications [66]. As exemplified, even application of the depletion approach followed by shotgun analysis did not allow the identification of the majority of low abundance proteins. Additionally, some issues concerning the depletion approach include: a) reproducibility, b) co-depletion of proteins of interest, c) requirement of higher amount of starting material or d) removal of only selected highly abundant proteins. Alternative, extensive fractionation can be performed. Recently, Atanassov et al. [67] described the combination of three separation strategies namely 1D-PAGE, pIEF (peptide isoelectric focusing) and RP-HPLC as an effective methods for reaching deeper proteome. Authors analyzed the nuclear extract from HeLa cells using the single separation methods (1D-PAGE and pIEF) as well as combined approach for the nuclear extract from HeLa cells. Increasing number of peptide and protein identifications were observed for the established three dimensional workflow (56228 peptides, 5260 proteins) in comparison to each individual approach (1D-PAGE-LC-MS/MS: 38557 peptides, 3540 proteins; pIEF-LC-MS/MS: 31113 peptides, 3945 proteins). Zhang et al. utilized a mix-bed ion-exchange chromatography (mixture of the strong cation and anion-exchange material) for protein separation prior to MudPIT analysis (multidimensional-protein identification technology) [68]. The workflow was tested using cell lysate from mammary tumor 4 T1. Using the classical MudPIT approach, 1292 proteins were identified, whereas application of 3D workflow enabled identification of 3084 proteins and covered 86% of identified proteins using classical MudPIT. Therefore, application of additional fractionation strategy can improve the proteome coverage. On the other hand, Zubarev et al. recently showed that the in-depth proteomic analysis can be performed using only one dimensional LC-MS/MS [69]. However, optimization is necessary, including sample preparation, chromatographic separation as well as MS analysis. The authors report that a 4 h procedure using a 50 cm column and MS/MS analysis enabled the identification of 37554 peptides corresponding to 4825 proteins (1% FDR at peptide and protein level). This covers around 50% of analyzed human malignant melanoma cell line (A375). Apparently, state-of-the-art MS-based platforms combined with optimized analytical workflow enable “deep” proteome coverage. Moreover, in comparison to the multidimensional procedures, the lower initial amount of starting material, shorter analytical time and cost-effectiveness are advantageous. In this part of review, we will summarize the current untargeted proteomics platforms for biomarker discovery along with the recent and representative examples of their application in bladder cancer biomarker discovery.

#### Gel-based proteomics platforms for biomarker discovery

Two dimensional gel electrophoresis (2-DE) followed by MS serves as a classical approach in analysis of differentially expressed proteins [70-72]. In this method, two separation steps are conducted namely isoelectric focusing (IEF) and SDS-PAGE. First, proteins are separated according to their charge in an immobilized pH gradient (IPG) and subsequently based on their molecular mass in a polyacrylamide matrix. Afterwards, protein spots are visualized and the signal intensity is used for (semi)quantitative analysis [73,74]. 2-DE enables separation of up to 10000 proteins [75] along with detection of protein isoforms [76,77]. Particularly, analysis of post-translational modifications including phosphorylation and glycosylation is of paramount importance, since their alteration is frequently related with pathological states. However, moderate reproducibility and limited detection for hydrophobic proteins (such as membrane), low abundance proteins, proteins above or below the pore size of the gel as well as proteins beyond the pH range of the IPG strips are shortcomings of 2-DE [78].

Difference gel electrophoresis (DIGE) is the recent advancement in traditional 2-DE [79]. In this approach three fluorescent cyanine dyes (Cy2, Cy3, and Cy5) of identical charge, similar molecular mass and different fluorescent properties, are used to label the proteins before separation [79,80]. The three samples: control, case and internal standard (combination of equal amounts of tested samples) are pooled after labeling and separated simultaneously. This reduces the number of gels as well as gel-to-gel variability, which is one of the major drawbacks of the classical approach [79,80]. Also, improved matching and more accurate quantification are achieved due to the presence of internal standard on all gels [80]. DIGE is more expensive and requires additional equipment like fluorescent scanner, but also offers higher sensitivity (0.5 fmol proteins) [80].

#### Gel-free proteomics

##### Shotgun proteomics

To bypass the limitations of the classical gel-based approach, efforts have been focused on the development of gel-free strategies. Shotgun or “bottom-up” proteomics is based on the analysis of native or protease derived peptides followed by sequencing with tandem mass spectrometry (MS/MS). Keeping in mind that the complexity of the sample is high, to improve the proteome coverage extensive/multidimensional fractionations has to be conducted prior to MS/MS analysis. Currently, different fractionation strategies are applied including mostly chromatography (ion exchange, reverse phase etc.) [68,69], IEF [66] or combination of these techniques [67]. This issue was described in the introductory part in this section.

In general, the large-scale proteomic analysis by MS/MS includes the following parts: 1) peptide ionization, 2) separation of precursor ions based on the mass-to-charge ratio, 3) fragmentation, 4) analysis of daughter ions, and 5) data analysis including identification and quantification [81]. Two major quantification approaches can be employed namely: label-based (metabolic or chemical labeling) and label-free (spectral counting and intensity-based analysis) approaches. Both approaches along with their advantages and limitations were extensively reviewed by DeSouza et al. [82]. Briefly, peptides from particular samples are labeled with different tags, mixed and analyzed by MS. In metabolic labeling (e.g. SILAC) heavy isotope amino acids are incorporated during protein synthesis, while in chemical labeling (e.g. Isobaric tag for relative and absolute quantification (iTRAQ), Isotope- coded affinity tags (iCAT) are applied after tryptic digestion. The mass shift introduced by the label is used to distinguish components derived from the different samples. The analytical variability on quantification is reported to significantly reduced in this experimental procedure in comparison the label-free approach where samples are measured separately, and differences in sample preparation as well as run-to-run variability can affect the results. Labeling is more expensive, since it requires isotope specific labeling reagents, and may also result in the introduction of artefacts, as well as a reduced coverage and dynamic range, due to the pooling.

Even though tandem mass spectrometry can result in high number of protein identifications, the false positive and false negative identifications are an inherent problem in shotgun experiments. Since shotgun proteomics is a peptide-based approach, the false protein identifications can occur as a result of incorrect assignment of fragmented ion spectra to peptide sequence as well as further inferring of protein identifications. Therefore, the false discovery rate is evaluated on peptide and protein level. Strategies to evaluate the false discovery rate for mass spectrometry results include searching against concatenated (chimeric database, composed from targeted and decoy database) [83] or decoy database [84], application of statistical models [85,86], or utilization of scoring system [87]. Methods for evaluation of assignment of peptides to protein sequences have been also described [88,89]. Of note: in general estimates of FDR are too optimistic [90], and the true FDR may be up to 10-fold higher.

##### CE-MS

Another interesting strategy that can be used in the biomarker discovery phase relies on analysis of the low molecular weight proteome, also defined as “peptidome”. For the identification of biomarkers on the peptidomics level, capillary electrophoresis coupled to mass spectrometry (CE-MS) has been widely applied [91-93]. Analytical performance of CE-MS was described recently and several issues including precision, stability, limits of detection, reproducibility and intra-variability were addressed [42]. The developments and applications of this platform in clinical proteomics were reviewed recently [94,95]. Briefly, small proteins and peptides are separated through an electric field according to their charge and size. Currently, separation in an uncoated bare fused silica capillary column at low pH is the best practical solution [95]. Different capillary coatings have been proposed to improve the analytical performance (robustness, resolution, reproducibility), however due to coating instability none of these techniques has been routinely used [96]. After electrophorethical separation, analytes are ionized (mostly by electrospray ionization (ESI) followed by MS analysis. Two coupling approaches are generally employed: sheathflow and sheathless interface. In general, all large-cohort studies are performed using the sheathflow approach, while sheathless interfaces currently are of limited robustness and reproducibility (as presented in the table from [95]). Detected peptides are characterized by the CE migration time, signal intensity and molecular mass. For identification, different platforms were tested e.g. CE-MALDI-TOF-TOF (Matrix-assisted laser desorption/ionization time-of-flight mass spectrometry), CE-ESI-QTOF (Quadrupole time-of-flight mass spectrometer) [97] and CE-Orbitrap [98]. The migration time in CE is dependent only on mass and the number of basic, neutral and polar amino acids [97]. This fact can be used to assign the peptide sequences obtained from LC-MS/MS (Liquid Chromatography) analysis to peptide mass that is detected by CE-MS [97].

#### Statistical data mining for proteomic biomarker discovery

Current proteomic approaches enable assessment of thousands of biomarker candidates. As aforementioned, at this stage a high false positive rate is observed [10]. Therefore, robust statistical analysis that allows the determination of “true” as well as promising candidates for further verification is of paramount importance [99]. Dakna et al. examined different statistical tests to discover valid biomarkers from proteomic data [100]. The non-parametric Wilcoxon test was found best suited for analysis of proteomic data: after validation in an independent sample set the highest percentage of valid biomarkers were detected. Additionally, the authors showed that adjustment for multiple testing is mandatory to derive potential biomarkers that can be verified in an independent test set.

Obviously, selection of candidates should not be based only on statistical significance, but also on the ability to fulfill a specific clinical need [43,100]. However, poor statistical design at the early experimental stage results in lack of statistical power to select relevant candidates, due to insufficient number of samples analyzed [43,100].

### B) Verification of biomarkers

Verification is mandatory to evaluate the findings obtained in the discovery phase. Although in discovery phase unbiased or untargeted approaches are applied to define a candidate or a set of candidate biomarkers, the verification phase represents a targeted approach for further evaluation of biomarkers sensitivity, specificity and predictive capabilities. More importantly, the verification is performed on the appropriate biological specimen that may be further used in clinical practice. In general, verification studies require larger patient populations than the discovery phase. Depending on the specific study design the analytical platform could be the same as in the discovery phase (CE-MS based classification) or vary with preferable assays to be either mass spectrometry based [101,102] or protein binding assays [103].

#### Protein binding assays

Protein binding assays include both the traditional immunoaffinity based ELISA (Enzymed- linked Immunosorbent assay) or other multiplex assays and Protein microarrays [104]. The most widely used technique for protein quantification is ELISA. The advantages of the method are speed, sensitivity and specificity, and compatibility with standard clinical laboratory equipment, so that it can be applied in clinical routine. The selectivity depends on the antibody that is applied and furthermore it has to be evaluated in the specimen of interest. The additional limitation of ELISA is that it cannot provide a simultaneous quantitative analysis of multiple potential biomarkers. In order to obtain quantitative data via parallel analyses for multiple antigens, Multiplex immunoassays have been developed. Protein microarrays are designed to print specific antibodies or antigens onto a support surface, generally a slide or membrane. A single sample is hybridized to the array. The captured antigens or antibodies are subsequently detected [105]. Assay platforms such as MULTI-ARRAY (Meso Scale Discovery), Bio-Plex (Bio-Rad Laboratories), have been applied for Cytokine detection, while regarding the renal injury a panel of 7 Biomarkers based on Antibody assays has been proposed as biomarkers with improved potential to assess renal function [106]. A major risk in the multiplex arrays is the increased cross-reactivity, due to the presence of multiple antibodies, which are normally applied as a mixture. To improve the assay specificity, Juncker and his collaborators have developed a number of innovative platforms with improved performance [107-109]. As a solution to avoid the mixing of the reagents, this group attempted the application of glass slides in a device called “snap chip”. The antibodies are immobilized in a multiple arrangement on a glass slide, where the sample is also applied [107]. The above group also introduced the use of gel captured antibodies in alginate droplets to increase the sensitivity of the detection. In this study in 2011, the assay was evaluated using 6 proteins, 3 already reported cancer biomarkers, as well as 3 cytokines (CEA, HER2, ENG and TNF-a, IL-8, MIP/CCL4 respectively) [108]. A new methodology based on the co-localization of the primary captured antibodies and the secondary detection antibodies is now proposed, namely ACM or Antibody Colocalization Microarray. When compared with the classical singleplex ELISA and conventional multiplex sandwich assays, ACM was proven to decrease the level of cross-reactivity. However, this technique could be more complex as it requires precision in the alignment [109].

In general, immunoassays are widely used in U.S. Food and Drug Administration (FDA) approved devices for cancer biomarkers, possibly also as a result of bias of the regulatory agencies towards conventional, but well characterized technologies. Many applications are reported, as reviewed by Fuzery et al. [8].

#### Mass spectrometric quantitative approaches

Apart from antibody-based technologies alternative methods for quantitative analysis and validation of potential biomarkers are quantitative MS methodologies, including the application of scanning techniques, such as multiple reaction monitoring (MRM) and other stable isotope labeling-based approaches such as SISCAPA (Stable Isotope Standards and Capture by Anti-Peptide Antibodies). The combination of high throughput capabilities of Mass Spectrometry, together with increased specificity and sensitivity that can be compared to immunoassays in some cases are the main advantages that make MS based applications very popular for quantitative validation studies [110]. MRM has the great advantage that an antibody is not required, but still awaits application in a clinical setting [111].

##### Multiple reaction monitoring

MRM is the extended version of Selected Reaction Monitoring (SRM) [112]. The analysis is focused only on biomolecules of specific masses, while all others are excluded. Higher specificity is achieved by the isolation of a specific precursor ion, collision-induced fragmentation and the subsequent detection of the specific product ion after fragmentation. Triple quadrupole instruments are typically employed for this approach [111,113,114]. A recent application in biomarker characterization in tissue specimens from patients with colorectal carcinoma was introduced (Hyperplex MRM). In this study a combination of a strategy for relative quantification such as iTRAQ was conducted with an mTRAQ approach for absolute quantification. This resulted in increased robustness of the MRM approach since 4 different samples were labeled simultaneously and in increased validity of the quantification since relative and total quantities of the biomarkers could be achieved [115]. Another variation of MRM methodology is a peptide immunoaffinity enrichment technique coupled with stable isotope dilution mass spectrometry, called SISCAPA [116]. In this technique, one or more selected tryptic peptides with unique sequences representing the target protein, the “proteotypic” peptides, are enriched using anti-peptide antibodies bound to Protein G. A stable isotope dilution (SID) method is applied as an internal standard by the use of a defined quantity of spiked stable isotope- labeled peptide of the same sequence in a pre-defined quantity. The relative quantification of the peptides is indicative of the protein concentration in the sample. In this assay, the sensitivity and specificity of antibody binding is combined to the versatility of MS, providing several advantages compared to the conventional immunoassays. Moreover, it provides the capability of analysis of multiple analytes in a single assay by combining antibodies in the enrichment step [117-119].

##### Pre-treatment strategies

In order to decrease the limit of detection, different pre- treatment strategies can be combined, like enrichment of the peptides of interest, sample pre-fractionation and depletion of the high abundance peptides. For targeted peptide enrichment, specific anti-peptide antibodies can capture the peptides of interest in the way it described above in the SISCAPA approach [120].

Many studies have reported the value of sample pre-fractionation and/or depletion. Kuhn et al. [121] first applied this strategy to characterize C-reactive protein in serum of patients with rheumatoid arthritis upon depletion of the 3 most abundant proteins in serum: albumin, immunoglobulin G, and haptoglobin. Yang et al. conducted two-dimensional solid-phase extraction as fractionation step prior to quantification of somatropin in plasma samples [122]. Keshishian et al. reported a 1000-fold improvement of limit of detection (LOD) upon depletion of seven high abundant plasma proteins by strong cation exchange chromatography [123]. A range of the values for the limit of quantification (LOQ) was between 1 and 10 ng/ml and coefficient of variation (CV) of 3-15% was estimated [123]. Employing the SISCAPA methodology, Kuhn et al. enriched for troponin-I and interleukin-33 in plasma samples to characterize these proteins as cardiovascular biomarkers [124]. To assess the inter-laboratory performance of immunoaffinity enrichment coupled to MRM- MS, Kuhn et al. designed an inter-laboratory study based on the quantification of 8 predefined peptides from S100A7, S100A8, S100A12, and IL1RN proteins [125]. Coefficient of variation was calculated for replicates analyzed by the same system (intra-laboratory) and across different laboratories (inter-laboratory) [125]. Overall inter-laboratory CV was estimated below 25 at the LOQ level. Inter-laboratory CV for immuno-MRM particularly, was calculated to be 14%, while intra-laboratory CV for immuno-MRM was 7%, respectively [125].

#### Data mining & statistical analysis

Depending on the quantitative approach that has been followed, different methodologies can be followed for peak integration, data analysis and downstream statistical evaluation [126,127]. For relative quantification or differential expression purposes, data normalization has to be performed prior to every type of comparison. A widely used approach especially when using label-free proteomics is the one described by Jantos-Siwy et al.[128], where endogenous stable and abundant peptides are used as internal standards. Another approach is SRMstats which can be applied to adjust for the median of the logarithmic values of the intensities obtained by the heavy isotope labeled peptides [129]. Variation caused by the analytical process or sample treatment may be corrected with the same methodology proposed by Johnson et al. for microarrays studies [130], where parametric and non-parametric formulas are applied, taking into account the mean intensity and variance in each sample [130].

### C) Bioinformatics platforms in clinical proteomics

Knowledge of biological mechanisms is helpful in the interpretation of proteomic results [131]. The application of computational techniques in analysing information associated with biomolecules on a large-scale platform has now been firmly established as a discipline in molecular biology encompassing a wide subject area from structural biology, genomics to gene-expression studies. Biological data at the omics level from transcriptomics to proteomics and metabolomic profiles are being produced at a very high rate [132,133]. For such a surge in data, computing science has become indispensable to biological research especially in handling large quantities of data and probing the complex dynamics observed in nature [134]. The main aims of bioinformatics include:

1) The organization of the data in a way to allow researchers access existing information and to submit new entries. Some of which include GEO [135], ArrayExpress [136] and Human Proteinpedia (http://www.humanproteinpedia.org/),

2) The development of new tools and resources for data integration and analysis, for which expertise in computational theory as well as a thorough understanding of biology is required. Such examples are interaction databases like IntAct, BioGrid and databases related to diseases like OMIM, Oncomine and metabolomics databases like HMDB.

3) The application of these tools in data analysis and interpretation of the results based on a biological meaningful manner, for instance web based tools like String and Cytocape for visualisation or AmiGO, KEGG, DAVID on the pathway level.

In particular for proteomics datasets, gene ontology and pathway annotations, as well as patient information should not only contain high confident data but should also be in a well-structured architecture to provide genuine data retrieval, coverage, and utility [137]. Some of the reliable protein/peptide and biological pathway resources used for proteomics profile processing in research and academic firms are described in Tables 1 and 2. After relying on annotated data sets from different databases (Table 2), the next steps are computational approaches in a systematic manner to analyse such integrated data. Computational approaches also provide means for inferring *in silico* and analysing changes in interactions and network dynamics [138]. Some of the computational tools for integrating proteomics datasets on a pathway level are:

1) Pathway analysis: KEGG [139], Ingenuity Pathway Analysis (http://www.ingenuity.com) MetaCore (http://host.genego.com/metacore.php)

2) Pathway mapping: Reactome [140], PathViso [141], BioCyc plugin [142]

3) Gene Ontology analysis: ClueGO [143], BiNGO [144], FuncAssociate [145]

4) Network analysis: GeneMania [146], DisGeNet [147], EnrichmentMap [148], NetAtlas [149], NetworkAnalyzer (http://med.bioinf.mpi-inf.mpg.de/netanalyzer/index.php) [150], KUPNetViz [151]

5) Interactome mapping: iRefScape [152], MiMI [153], PanGIA (http://prosecco.ucsd.edu/PanGIA/), BioNetBuilder [154], Bisogenet [155], FunNetViz (http://www.funnet.ws/)

6) Metabolomics analysis: IDEOM [156], MAVEN, MetaCore, Beilstein, mzMatch [157]

**Table 1:** List of reliable protein and peptide databases

**Databases for protein/Peptide data repository**	
**Protein/Peptide Database**	**Website/Link**
UniProt/Swiss Prot	http://www.uniprot.org/
Proteomics Identifications Database	http://www.ebi.ac.uk/pride/
MEROPS	http://merops.sanger.ac.uk/
PepBank	http://pepbank.mgh.harvard.edu/
PeptideAtlas	http://www.peptideatlas.org/
ProteinProspector	http://prospector.ucsf.edu/prospector/mshome.htm
MassMatrix	http://www.massmatrix.net/mm-cgi/home.py

**Table 2:** List of highly cited pathway databases for proteomic applications

**Most cited repositories for biological pathways**		
**Pathway databases**	**Biological pathway**	**Website/Link**
Reactome KnowledgeBase	Signal Transduction Pathway	http://www.reactome.org
BioCarta Pathway Diagrams	Signal Transduction Pathway	http://www.biocarta.com/genes/index.asp
Pathway Commons	Signal Transduction Pathway	http://www.pathwaycommons.org/pc/
Protein ANalysis THrough Evolutionary Relationships	Signal Transduction Pathway	http://www.pantherdb.org
Protein Lounge	Signal Transduction Pathway	http://www.proteinlounge.com
WikiPathways	Signal Transduction Pathway	http://wikipathways.org/
Transcription Factor encyclopedia	Regulatory Pathways	http://www.cisreg.ca/cgi-bin/tfe/home.pl
Transcription Regulatory Regions Database	Regulatory Pathways	http://wwwmgs.bionet.nsc.ru/mgs/gnw/trrd/
A Public Database of Transcription Factor and Regulatory Sequence Annotation	Regulatory Pathways	http://www.pazar.info/
Homo Sapiens Comprehensive Model Collection (HOCOMOCO)	Regulatory Pathways	http://autosome.ru/HOCOMOCO/index.php
Transcription Factor Database	Regulatory Pathways	http://www.gene-regulation.com/index2.html
Human Protein Reference Database	Protein-Protein Interactions	http://www.hprd.org/
Human Annotated and Predicted Protein Interaction Database	Protein-Protein Interactions	http://bio.informatics.iupui.edu/HAPPI/
Biomolecular Interaction Network Database	Protein-Protein Interactions	http://bond.unleashedinformatics.com/
Molecular Interaction Database	Protein-Protein Interactions	http://mint.bio.uniroma2.it/mint/
Biological General Repository for Interaction Datasets	Protein-Protein Interactions	http://thebiogrid.org/
Search Tool for the Retrieval of Interacting Genes/Proteins	Protein-Protein Interactions	http://string.embl.de/

#### Applications of systems biology – disease diagnosis and treatment

Network based approaches to human diseases appear to have enormous potential in biological and clinical applications. To better understand the effects of cellular mechanisms on disease progression, identifying proteins and pathways that are related to disease may offer better targets for drug development. These advances may also lead to the selection of better and more accurate biomarkers that are associated with diseases and help with disease classification. Current systems-based approaches focus on identifying pathways that may be used to subtype a disease and develop treatments for individual disease groups. Network modules have been used to predict patient survival, metastasis, invasion, drug response etc. [158-164]. For this purpose, a well characterised group of samples is required related to a disease subtype/stage, for example cancer metastasis to search among specific networks or so called sub-networks for potential biomarkers that enable disease classification [165]. Additionally, systems analysis may provide with insights in the molecular mechanisms underlying the diseases. This may be highly valuable in drug development by indicating correlation between the response to a drug and the responders’ molecular background. An example of such an approach is the study by Chu and Chen, where a protein-interaction network was applied to investigate drug targets related to apoptosis [166].

### D) Validation of biomarker candidates

The pivotal objective of the validation phase is to evaluate the clinical utility of the biomarker candidates [9]. Validation has to be performed in an independent, sufficiently large sample set also reflecting the heterogeneity of targeted population. This is mandatory also since the diagnostic accuracy is often generally overestimated in the model established in training set (groups of individuals used for discovery of biomarkers and development of the model) [52]. To demonstrate the clinical utility, validation studies have to be driven by the specific context of use and targeted population, since depending on the clinical needs the biomarker has to fulfill different requirements regarding clinical performance (i.e. sensitivity and specificity). The accuracy of individual biomarker or biomarkers panel performance can be assessed by the ROC (receiver operating characteristics) analysis [167]. ROC curve represents a plot of true-positive rate (sensitivity, percentage of cancer patients who tested positive for biomarkers) versus false positive rate (FPR, percentage of healthy subject classified as having disease). Whereas specificity is defined as 1-FPR. In this method the area under the curve (AUC) is used as an indicator of the biomarker performance regarding the ability to distinguish between control and patients affected by disease. It is of paramount importance to take into account the false positives and false negatives in order to establish an optimal classification threshold at desired specificity and sensitivity level. Biomarkers utilized for screening should reveal high sensitivity and, frequently even much more important, a low level of false positives. On the other hand, specific diagnostic tests require high positive predictive values (PPV, percentage of diseased patient among all positive test result). Due to the fact that sensitivity and specificity do not provide the information about probability of disease occurrence, disease predictive values have to be assessed i.e. (PPV, PPN). However, these values are dependent on disease prevalence and can only be assessed in prospective studies [8]. Collectively, regardless of the clinical use, consequences from false positive and false negative cases have to be always considered as a benefit-to-harm ratio. Ultimately, application of novel biomarker/biomarkers panel has to improve the outcome. A striking example of an unfortunate development is the prostate specific antigen (PSA) test. The applicability of this test for prostate cancer screening arose controversy in medicine [168]. It has been claimed that decrease of mortality for prostate cancer is the major benefit of PSA-based screening. However, Andriole et al. reported that the screening does not reduce the number of deaths for prostate cancer after 13-years follow up [169]. Moreover, the false-positive results (and they represent the majority in this case) have harmful consequences including invasive biopsy as well as following complication, overdiagnosis and overtreatment of disease [168].

Varied challenges are encountered at the validation stage including e.g. 1) samples quality and availability, 2) funding and 3) requirements of regulatory agencies [170]. Due to these facts, validation is a bottleneck in the biomarker development process [170]. In addition, the scientific reward in validation is moderate: validation studies are generally rejected in high impact journals. To also ensure robustness, validation should be performed in a multi-center study [52], and selected cohorts have to represent the population targeted with the biomarker. Bearing in mind that most promising candidates have to be tested in hundreds or even thousands of samples, validation requires quantitative, robust, (multiplex) and high throughput methodology. Not all of the platform applicable at previous stages can fulfil these requirements. Therefore, changing the platform can be necessary: biomarkers discovered using gel-based approach cannot be further validated by the same techniques, mainly due to its limited throughput. On the other hand, CE-MS serve as a good example of a technique, which can be applied for all biomarker development phases [91-93]. High reproducibility, high throughput and cost-effectiveness are reported [42]. These characteristic along with the need for developing biomarker panels, makes CE-MS an attractive platform for biomarker development.

Currently, antibody-based approaches are considered as the gold standard in clinical application, mostly since this is a technology well known to everybody. However, application of immunoassays is often hampered by the lack of high-quality antibodies. Additionally, the ELISA assay enables detection of a single antigen, a drawback for the validation of biomarker panels. Moreover, the low-dynamic range and high cost of development of ELISA based assays indicate a moderate utility of this approach in large scale validation studies. To bypass some of the limitations multi-analyte immunoassays have been applied [171] including planar array [172] and micro-bead assays [173,174]. In planar array, different antibodies are spotted on a flat surface, whereas in a second type, antibodies are immobilized to varied micro-beads [172]. Recently, Fu et al. [175] compared the analytical performance of five currently used multiplex immunoassays in the context of their application for validation of biomarkers (particularly cytokines) in serum. The MULTI-ARRAY (planar assay) and Bio-Plex (magnetic beads) are characterized by better performance than other tested multiplex assays i.e. A2 (Beckman Coulter), FAST Quant (Whatman Schleicher & Schuell BioScience), and FlowCytomix (Bender MedSystems), but this is also dependent on analyzed biospecimens (serum or purified cytokines) [175]. Application of multiplex assays enables high-throughput quantitative analysis and uses less sample volume. On the other hand, development of novel multiplex immunoassays is a challenging task. Currently, antibodies are commonly used as capture ligands, but aptamer ligands (oligonucleotides) may offer an alternative novel approach [176,177]. A promising alternative are MS-based (typically MRM) approaches, as described in details in previous section. After establishing clinical utility (which equals significant improvement over the current state of the art) [16], further assessment of analytical performance is required. In this case, the following issues have to be addressed: detection and quantification limit, precision, stability of analyte, specificity, interfering compounds etc. This topic was covered recently by Fuzery et al. [8] and it is beyond of the scope of this paper to review this issue in detail.

## Application of proteomics approaches in BCa biomarker discovery

Proteomic approaches have been applied at all stages of biomarker discovery workflow. This includes untargeted platforms for biomarker identification (gel-based and gel-free) [70,71,178-182] and targeted platforms for further verification and validation of biomarker candidates (MRM, multi-analyte assays) [106,108,115,183,184]. Additionally, to improve proteome coverage and identify low abundance protein, enrichment strategies have been also applied e.g. immobilized metal affinity (IMAC) [179,182] dual-lectin chromatography [185], or peptidomics approaches [92,93]. Bladder cancer is the second in incidence and mortality malignancy of the genitourinary system. At initial diagnosis, the majority of patients (75%) exhibit non-muscle invasive cancer (pTa, pT1, pTis), whereas the rest belongs to muscle invasive disease (pT2, pT3, pT4) [22]. The invasive phenotype results in significant decrease of the survival rate [22]. Additionally, high recurrence rate and cancer progression impose the requirement for lifelong monitoring of patients after treatment. Up to date, the gold standard for clinical diagnosis includes invasive cystoscopy and non-invasive voided urine cytology with limited sensitivity for detection of low grade tumors [186]. Although some tests have been approved by FDA (e.g. NMP-22, BTA-TRAK, uCyt+), they seem to have no clinical utility [187-189]. Therefore, there is an urgent clinical need for application of novel non-invasive tests for early detection, patient monitoring and stratification. A vast number of potential biomarkers have been discovered using proteomics as well as genomics approaches. The detailed description of currently available bladder cancer biomarker candidates is beyond the scope of the manuscript and this topic was recently reviewed [190,191]. To give an overview on the current status of BCa proteomic biomarkers, the representative examples along with study design and potential clinical utility are described below and summarized in Table 3.

**Table 3:** Representative examples of BCa biomarker candidates identified by proteomic approaches

***Biomarker identification; Biomarker candidate/panels***	***Verification/Validation***	***Regulation***	***Potential clinical value; Biomarker performance***	***Ref.***
***Gel-based approaches***				
***2DE*, n=24**				[178]
Tissue:	**Western Blot**	↑ in both NMIBC and MIBC;	Predict cancer progression	
6 normal urothelium, 9 NMIBC, 9 MIBC	**Immunohistochemistry, n=24**	↑ phosphorylation level of cofilin in BCa tissue samples (most prominent in MIBC).	Lack of evaluation of biomarker performance.	
**Cofilin**	*For both experiments, the same material was used as in the discovery phase.*			
***IMAC, 1-SDS-PAGE*, n=35**	**Western Blot**			[182]
Urine:	Aminopeptidase N, n=108	Aminopeptidase N	Biomarker for cancer aggressiveness	
Two pools from NMIBC, n_1_=9, n_2_=7	Myeloblastin, n=97	↑ in MIBC		
Two pools from MIBC, n_3_ = 9, n_4_=10	**ELISA**	Myeloblastin, Profilin 1	Lack of evaluation of biomarker performance.	
**Aminopeptidase N, Myeloblastin,**	Profilin-1, n=82	↓ in MIBC		
**Profilin-1**				
	**Western Blot,** 8 BCa cell line models	↑ in BCa cases, association with stage		[181]
***DIGE*, n=14**	**Tissue microarray, n=292**		Diagnosis, staging, outcome prognosis	
Urine:	Primary urothelial cell carcinoma		**Detection of BCa:**	
7 BCa (positive cytology), 7 controls (negative cytology)	**ELISA, n=80**			
**Reg- 1**	Urine:		81.3% sensitivity	
	32 BCa (positive cytology), 48 Controls		81.2% specificity	
	(negative cytology)			
***Gel-free approaches***				
	**ELISA, n = 166**			[179]
	Urine,			
***LC-MS/MS*, n=20**				
Urine:	For H2B: n=147,	↑ level of H2B with cancer stage in urine and tissue samples	Prediction of disease progression, discrimination of tumor stages	
Benign (n=5), pTa, pT1 (n=10), pT2+ (n=5)	For NIF-1: n = 158			
	*In both groups urine from benign, NIMB (Ta, Ta) and MIBC (T2+) were included.*			
**histone H2B, NIF-1**				
		↓ level of NIF-1 with cancer stages (not agreement with urinary level)	Lack of evaluation of biomarker performance.	
	**Immunohistochemistry, n=32**			
	pTa, pT1, n=23, pT2+ n=9			
***iTRAQ*, n=12**	**Immunohistochemistry, n=303**	↑ in 4/6 BCa samples in comparison to control (iTRAQ);	Prediction of disease progression	[180]
Tissue:				
6 bladder cancer tissues (4 NMIBC, 2 MIBC) and paired normal tissues;				
		Inverse correlation to stage and histological grade progression (immunohistochemistry)	Lack of evaluation of biomarker performance.	
**DDX39**				
***CE-MS*, n=248**	**CE-MS, n=130**	↓ regulated in MIBC in comparison to NMIBC	**Prediction of MIBC:**	[92]
Urine:	Urine,		81% sensitivity	
127 BCa patients, 121 Controls	*Test set:* 68 NMIBC and 62 MIBC		57% specificity	
**4 polypeptide panel**				
***CE-MS*, n=79**	**CE-MS, n=366**	Varied; 10 peptides ↑ in BCa;		[93]
Urine:	Urine,	12 ↓ in BCa in comparison to control	**Detection of BCa:**	
46 BCa patients, 33 Controls	*(Test set includes healthy controls, patients with non-malignant and malignant urological disorders)*		100 % sensitivity	
**22 polypeptides panel**			73% specificity	

### Gel-based proteomics

Chung et al. detected by 2-DE elevated levels of cofilin in BCa tissues vs. control urothelium. In total, 24 samples were analyzed resulting in identification of 12 differentially expressed proteins. The up-regulation of cofilin in BCa tissue specimens was confirmed by Western Blot and immunohistochemistry. Additionally, an antibody specific for phosphoylated Ser-3 of cofilin revealed elevated phosphorylation in BCa samples, especially in muscle-invasive BCa. In parallel, in vitro studies showed decreased EGF-induced migration in cofilin knock-down T24 cells. Collectively, both expression and phosphorylation of cofilin may be involved in BCa aggressiveness [178]. In another study, the urinary proteome was investigated in order to detect biomarkers for aggressive BCa. Zoidakis et al. analyzed urine samples from NMIBC and MIBC patients using enrichment by IMAC [182]. Collected enriched fractions were separated using 1D-SDS PAGE followed by in-gel digestion. Protein identification was performed using LC-MS/MS analysis. The study revealed aminopeptidase N, profilin-1 and myeloblastin as potential biomarker candidates. Further confirmation by Western blot or ELISA was conducted for selected proteins. Aminopeptidase N found to be down-regulated in MIBC, whereas Profilin-1 and myeloblastin were up-regulated in invasive cancer [182]. Orenes-Pinero has applied differential gel electrophoresis to investigate the urinary proteome of BCa patients (n?=?7, positive cytology) and controls (n?=?7, negative cytology) [181]. Differentially expressed proteins were identified by peptide mass fingerprinting using MALDI-TOF MS, including Regenerative protein (Reg-1), cytokeratins 1, 2 and 10, T-cell surface protein CD5 and prefoldin. Among these, only cytokeratin 1 was down-regulated in urine from BCa patients. Western blot analysis of eight bladder cancer cell line models (from non-invasive to metastatic) indicated the correlation between the levels of the proteins identified by proteomics and cancer progression. Quantitative analysis of urinary Reg-1 was evaluated by ELISA (n?=?80) and used for evaluation of diagnostic accuracy. Sensitivity and specificity at the level of 0.0038 ng/mL were 81.3% and 81.2%, respectively.

### Gel-free proteomics

The shotgun approach followed by labeling or label-free quantification has also been widely implemented in biomarker discovery research. Frantzi et al. described urinary histone H2B and Zinc-finger 335 (NIF-1) as a potential progression marker for BCa. Urine from benign (n?=?5), non-invasive (n?=?10) and invasive cases (n?=?5) was enriched by IMAC and native peptides were analyzed by LC-MS/MS. It total, 1845 peptides were detected (638 precursor proteins). Differential regulation of histone H2B and NIF-1 were verified further by ELISA (urine, n?=?166) and immunohistochemistry (tissue samples, n?=?32) [179]. Apart from the label-free approach, labeling techniques have been also employed to discover biomarkers for BCa. Kato et al. used iTRAQ labeling to compare the proteome from bladder carcinoma urothelium (n?=?6) with paired normal tissues (n?=?6) [180]. 493 proteins were identified including 15 up-regulated proteins in cancer cases in comparison to adjacent normal samples (e.g. DDX39, B-cell receptor-associated protein 31, chaperonin containing TCP1, FK506 binding protein 4, S100 calcium binding protein A1). Immunohistochemistry (n?=?303) was used to verify the findings for protein which have not been previously evaluated. However, actin-related protein 3 homolog B was not verified, since the antibodies were not commercially available. This example indicates one of the drawbacks for application immune-based assays for protein verification: lack of specific antibodies. Authors found decreased expression of DDX39 with higher cancer stage and grade. In addition, low expression level of DDX39 significantly correlates with disease progression. Further functional analysis using siRNA assay was performed in bladder cancer cell line (T24). As a result, an increased invasion ability of cells transfected with si-DDX39 compared to control was observed. Therefore, reduced expression of this protein may serve as a biomarker to predict disease progression [180].

The search of potential biomarker candidates can be performed also at the peptide level. Briefly, peptidomic profiling was used for detection of urothelial carcinoma [93] as well as for prediction of MIBC [92]. In all these cases, biomarker panels were developed after analysis of a training set and further validated in independent test sets. CE-MS analysis was conducted for the discovery and initial validation phase. In a first study, Theodorescu et al. developed a 22 polypeptides panel for diagnosis of urothelial carcinoma [93]. The limited specificity (73%) was obtained in the test set (varied genitourinary disorders), whereas the sensitivity remained high (100%). Moreover, authors also advocated the application of additional discriminatory panels (e.g. non-malignant disease vs. urothelial carcinoma) can increase the specificity level [93]. In a second study, a 4 polypeptides panel (fragments of membrane associated progesterone receptor component I, uromodulin, collagen α-1 (I), Collagen α-1 (III)) was reported by Schiffer et al. and enabled detection of MIBC with sensitivity of 81%. However, limited specificity was obtained (57%) [92]. Along the same avenue of research, reanalysis of existing and newly collected BCa peptidomics data is currently under investigation in our lab (n?=?608 samples, 304 controls and 304 BCa patients). Several of previously detected peptides were confirmed as well as novel potential biomarkers were reported [95]. These data are currently rigorously verified in large, prospectively collected cohorts in the EU-funded BCMolMed (http://www.bcmolmed.org) project.

To summarize, the number of detected biomarker candidates depends on utilized analytical platform at biomarker discovery stage e.g. 2-DE enables identification of over a dozen candidates, whereas this values can increased up to hundreds for MS-based approaches. In most cases immune-based assays were used to verify presence of selected protein. Only for the peptidomics markers, CE-MS was applied for all phases. However, in most cases only initial verification of detected biomarkers was conducted, but appropriate vigorous validation in a sufficiently large population is still outstanding. Therefore, in order to establish robust and accurate biomarker/panel of biomarkers, further validation has to be performed, driven by the clearly defined context of use and cohorts representing the targeted population. Also, apart from the peptidomics studies, only single proteins were used to evaluate the diagnostic accuracy, whereas a combination of biomarkers candidates may lead to substantial improvement of biomarker performance.

## Conclusions

Cancer is considered as a disease with high heterogeneity, increased incidence and mortality rates with a serious social and economic burden. The benefits from application of robust and accurate biomarkers in cancer management might result in significant improvement of clinical outcome via detection of cancer at early stages. An improvement in the therapeutic strategies based on the prognosis of the treatment response is also anticipated [192]. Multiple advances have been achieved regarding the proteomic technology that can be applied in major parts of the biomarker development: identification, verification and validation. Along the way of recent achievements in untargeted MS-based proteomic approaches, as well as targeted quantification proteomic strategies, the number of potential proteomic biomarkers has rapidly increased, as also indicated by the biomarkers candidates related to BCa summarized in this article [193-196]. However, this apparent progress has not triggered successful implementation of novel biomarkers into clinical practice. Therefore, as pinpointed above, critical issues related to biomarker development should be taken into account to raise the awareness about difficulties encountered in the process. Shortcomings hampering the biomarker implementation include difficulties related to the definition of context of use, proper study design (selection of patients, statistical design), samples availability along with poor clinical characteristics, high sampling variability due to the lack of application of standardized protocol as well as the application of inappropriate statistics. Clinical implementation of biomarkers is complex and requires collaborative efforts between researchers from different fields and clinicians. In order to facilitate the translation into clinical utility and benefit for patients, various guidelines have been established to guide scientists in this endeavor [11,197].

Based on the literature published, it appears that numerous proteomic biomarkers do exist that will likely result in a substantial improvement of the current clinical situation [193,194,198].

Regarding Chronic Kidney Disease (CKD), a urinary biomarker model based on a panel of 273 peptides, as established after CE-MS analysis has been already well investigated in the context of early diagnosis of CKD. Good et al. [199] first proposed the above 273 biomarker model, reporting an AUC (area under the curve) value of 0.96 upon independent validation in an independent blinded cohort of 109 CKD samples and 34 urine samples from normal individuals [199]. The same performance (AUC of 0.96) was presented in a follow up study for the same model, using a multicentric validation approach including 137 urine samples (62 CKD patients and 75 normal controls) [200]. Importantly, Zurbig et al. [201] further evaluated the CKD273 peptide marker model for its diagnostic utility in a longitudinal study, where 316 urine samples were employed, including patients with diabetes type 1 and 2 [201]. In this study, the above model was able to predict the progression of normoalbuminuria to macroalbuminuria 5 years before onset, while the AUC value was estimated at 0.93, increased compared to the routinely used urinary albumin whose reported performance is 0.67 [201]. Finally, Andersen et al. [202] applied the CKD273 for characterization of the renoprotective treatment outcome in hypertensive type 2 diabetic patients treated with Ibersartan. In this study, urine samples were collected from patients undergoing Ibersartan treatment in two timepoints before and two years after treatment [202]. The changes in the peptide pattern of the treated patients are indicative of the possible utility of this model -and such proteomic biomarker approaches in general-, in the monitoring of the patients response to drug treatment [202]. The CKD273 classifier is currently been implemented in a Multicentric European Trial, called PRIORITY where 3280 patients with diabetes type 2 are employed.

Another CE-MS derived peptide biomarker approach with increased potential to be implemented as a routine test for diagnosis of cholangiocarcinoma (CC) has been also described [203]. Due to the demanding clinical need for early detection of cholangiocarcinoma that increases the curative potential of a therapeutic treatment, Lankisch et al. [203] first proposed a peptide classifier as established by interpretation of CE-MS data derived from bile proteomic analysis [203]. Two models were presented enable to distinquish between patients with choledocholithiasis and malignant lesions as well as between cholangiocarcinoma and primary sclerosing cholangitis (PSC), a risk factor for cholangiocarcinoma. After independent validation, the first model was found able to distinguish between patients with gallstones and malignant lesions with sensitivity of 93% and specificity of 86%, while the second model classified PSC cases and CC cases with a sensitivity of 84% and specificity of 78%. Following a similar approach, Metzger et al. [204] introduced a urinary based peptide classifier in a follow up study, where the specific aim was the investigation of a non-invasive urinary test for early diagnosis of cholangiocarcinoma. In this case, an AUC value of 0,87 was reported with 83% sensitivity and 79% specificity, after validation in a cohort of 123 patients [204].

Based on the literature available, it appears that clinically useful proteomic biomarkers can be identified, and also validated, employing the technologies available today. Hence, unravelling this potential benefit would “only” require analysis of a sufficient number of samples using appropriate technologies. Assuming the availability of the required funds, the only limitation is the availability of samples. This major problem has not successfully been tackled by the generation of biobanks, these generally do not proved the support anticipated [197].

However, as recently also suggested Vlahou [17], combining efforts and testing multiple biomarkers in the same samples may be the most promising approach. Another hurdle in bringing the benefits to the patients apparently are the requirements by regulatory agencies, and cost as well as the reluctance of the public health systems to accept novel and beneficial approaches in medicine. Here, initiative from the relevant clinical professional societies and patients groups may be needed in combination with simplification and improvements of regulatory requirements, to enable timely implementation of highly beneficial developments to improve medicine and patient care.

## Abbreviations

2-DE: Two dimensional gel electrophoresis; ACM: Antibody colocalization microarray; AUC: Area under the curve; BCa: Bladder Cancer; CC: Cholangiocarcinoma; CE: Capillary Electrophoresis; CKD: Chronic kidney disease; CV: Coefficient of variation; DIGE: Difference gel electrophoresis; ELISA: Enzymed- linked Immunosorbent assay; ESI: Electrospray ionization; FDA: U.S. Food and Drug Administration; iCAT: Isotope- coded affinity tag; IEF: Isoelectric focusing; IMAC: Immobilized metal affinity chromatography; IPG: Immobilized pH gradient; iTRAQ: Isobaric Tag for relative and absolute quantification; LC: Liquid chromatography; LIMS: Laboratory informatics management applications; LOQ: Limit of quantification; MALDI: Matrix-assisted laser/desorption ionization; MIBC: Muscle invasive bladder cancer; MS: Mass spectrometry; MS/MS: Tandem mass spectrometry; NMIBC: Non-muscle invasive bladder cancer; NPV: Negative predictive value; PPV: Positive predictive value; PSC: Primary sclerosing cholangitis; QTOF: Quadrupole time-of-flight mass spectrometer; SILAC: Stable isotope labeling by amino acids in cell culture; SID: Stable isotope dilution; SISCAPA: Stable Isotope Standards and Capture by Anti-Peptide Antibodies; SRM/MRM: Single/Multiple reaction Monitoring.

## Competing interests

Maria Frantzi and Akshay Bhat are employed by Mosaiques Diagnostics.

## Authors’ contributions

MF, AL and AB performed the literature review and contribute equally to the drafting of the manuscript. All authors read and approved the final manuscript.

## Authors’ information

All the authors namely MF, AB, AL are a PhD students involved in the European BCMolMed program (http://www.bcmolmed.org). The major goals of the project are identification of novel biomarkers related to bladder cancer invasion, verification of existing biomarkers as well as integration of data using system biology approach.
